# Indoor Radon and Incident Heart Failure Among Postmenopausal Women

**DOI:** 10.1001/jamanetworkopen.2026.42391

**Published:** 2026-11-05

**Authors:** Shu Chen, Cuiran Shi, Jason M. Collins, Ayesha K. Ekanayaka, Carson Mowrer, James D. Stewart, David Y. Huang, Matthew A. Allison, Charles B. Eaton, Bernhard Haring, Michael J. LaMonte, Matthew S. Nudy, Alexander P. Reiner, Gary G. Schwartz, David Z. Shou, Lesley F. Tinker, Mark R. Williamson, Richard L. Smith, Eric A. Whitsel

**Affiliations:** 1 Department of Statistics and Operations Research, University of North Carolina, Chapel Hill; 2 Department of Biostatistics, University at Buffalo, State University of New York; 3 Department of Epidemiology, Gillings School of Global Public Health, University of North Carolina, Chapel Hill; 4 Department of Biostatistics, Gillings School of Global Public Health, University of North Carolina, Chapel Hill; 5 Department of Neurology, School of Medicine, University of North Carolina, Chapel Hill; 6 Department of Family Medicine, University of California San Diego, La Jolla; 7 Department of Family Medicine, Warren Alpert Medical School and Department of Epidemiology, School of Public Health, Brown University, Providence, Rhode Island; 8 Department of Medicine IV and Karl Landsteiner Institute for Cardiovascular Research, Clinic Hietzing, Medical University of Vienna, Vienna, Austria; 9 Klinik für Innere Medizin III and Homburg Institute of CardioRenalMetabolic Medicine (HOMICAREM), Saarland University, Homburg, Saarland, Germany; 10 Department of Epidemiology & Population Health, Albert Einstein College of Medicine, Bronx, New York; 11 Department of Epidemiology and Environmental Health, School of Public Health and Health Professions, University at Buffalo; 12 Division of Cardiology, Penn State Health Milton S. Hershey Medical Center, Penn State College of Medicine, Hershey, Pennsylvania; 13 Division of Public Health Sciences, Fred Hutchinson Cancer Center, Seattle, Washington; 14 Department of Epidemiology, University of Washington, Seattle; 15 Department of Population Health, School of Medicine & Health Sciences, University of North Dakota, Grand Forks; 16 Department of Radiology, Walter Reed National Military Medical Center, Bethesda, Maryland; 17 Department of Medicine, School of Medicine, University of North Carolina, Chapel Hill

## Abstract

**Importance:**

Evidence has shown an association of radon exposure with heart failure risk factors, but whether there is an association with heart failure remains unclear.

**Objective:**

To assess whether indoor radon concentrations are associated with incident heart failure among US postmenopausal women.

**Design, Setting, and Participants:**

This prospective cohort study used data from the Women’s Health Initiative (WHI) observational study and clinical trials. Participants were postmenopausal women from 40 US clinical centers who were aged 50 to 79 years and did not have self-reported heart failure at enrollment or randomization (September 1993 to December 1998) but had physician adjudication of heart failure during follow-up through February 17, 2024.

**Exposure:**

Geocoded participant address–based assignment of county-level, US Environmental Protection Agency–categorized indoor radon concentration (<2, 2-4, or >4 picocuries [pCi]/L).

**Main Outcome and Measure:**

Incident hospitalization for acute decompensated heart failure was ascertained through physician adjudication of medical records. Hazard ratios (HRs) for heart failure by indoor radon concentration were estimated with Cox proportional hazards regression models.

**Results:**

Among 43 188 postmenopausal women (mean [SD] age, 63 [7] years), 36% were exposed to a radon concentration less than 2 pCi/L; 41%, to a concentration of 2 to 4 pCi/L; and 23%, to a concentration of more than 4 pCi/L. Over a mean (SD) follow-up of 16.2 (8.4) years, 3446 incident heart failure events occurred. The fully adjusted HR for heart failure was 1.19 (95% CI, 1.07-1.33) at more than 4 pCi/L vs less than 2 pCi/L, changing little in sensitivity analyses. The HR was higher for heart failure with reduced ejection fraction (1.24; 95% CI, 1.04-1.49) and heart failure mortality (1.37; 95% CI, 1.12-1.69) at radon concentrations more than 4 pCi/L vs less than 2 pCi/L, but these HRs were not different from the null at concentrations of 2 to 4 pCi/L vs less than 2 pCi/L. The HR at concentrations greater than 4 pCi/L approximated that of a 16–mm Hg increase in systolic blood pressure or an increase of 4.8 in body mass index (calculated as weight in kilograms divided by height in meters squared).

**Conclusions and Relevance:**

In this cohort study of US postmenopausal women, higher indoor radon concentrations were associated with an increased risk of incident heart failure, suggesting that radon may be a modifiable environmental determinant of heart failure.

## Key Points

**Question:**

Is risk of incident, physician-adjudicated heart failure associated with exposure to indoor radon among postmenopausal women in the US?

**Findings:**

In this cohort study of 43 188 postmenopausal women, indoor radon concentrations greater than 4 vs less than 2 picocuries per liter were associated with a significantly higher risk of heart failure, heart failure with reduced ejection fraction, and heart failure mortality.

**Meaning:**

In this study, higher indoor radon concentrations were associated with an increased risk of incident heart failure among postmenopausal women.

## Teaser

This cohort study of data from the Women’s Health Initiative examines associations between exposure to indoor radiation at various concentrations and risk of incident heart failure among postmenopausal women in the US.

## Introduction

Heart failure remains a leading cause of morbidity, mortality, and health care expenditure among women in the US,^1^ despite progress in managing established risk factors. Heart failure prevalence continues to rise, underscoring the value of identifying other accurately measurable and structurally modifiable risk factors for it. Radon, a colorless, odorless radioactive gas produced by the decay of uranium in soil and rock, accumulates indoors through soil-gas infiltration. Recent data suggest that radon concentrations have increased in North American homes,^2^ with approximately one-quarter of the US population now residing in areas exceeding the Environmental Protection Agency (EPA) action level of 4 picocuries (pCi)/L.^3^ As the largest natural source of ionizing-radiation exposure, radon has long been recognized as the second leading cause of lung cancer,^4^ yet more recent evidence, including that from repeated-measures studies and large US population-based cohorts, has also shown association of indoor radon and radon-related ambient-particle radioactivity with heart failure risk factors (eg, diabetes, hypertension, myocardial infarction, and stroke)^5,6,7,8,9,10,11^ and mortality among patients with heart failure.^12^ However, to our knowledge, the association of radon with incident, physician-adjudicated heart failure has not been characterized. We therefore investigated the association between indoor radon and incident heart failure in a large, prospective cohort of US postmenopausal women.

## Methods

### Study Population

We conducted a prospective cohort study among Women’s Health Initiative (WHI) observational study and clinical trials participants from 40 US clinical centers who were followed up for physician-adjudicated heart failure outcomes. The participants were aged 50 to 79 years at baseline (September 1993 to December 1998) and included Black or African American women, Hispanic or Latina women, and all women randomized in the hormone therapy trial.^13^ Among them, analyses included those without a self-reported history of heart failure at baseline who had follow-up for adjudicated heart failure outcomes through February 17, 2024 (eFigure 1 in Supplement 1). The WHI protocol was approved by the Fred Hutchinson Cancer Center institutional review board, and all participants provided written informed consent for study participation, review of their medical records, and use of the resulting information in deidentified data analyses. The current WHI Radon Risk Investigation study (WHI Risk It?) was separately reviewed and approved by the WHI ancillary studies committee, WHI observational studies monitoring board, WHI presentations and publications committee, and Wake Forest University Health Sciences institutional review board. This study followed the Strengthening the Reporting of Observational Studies in Epidemiology (STROBE) reporting guideline.

### Exposures

We defined the primary exposure as the indoor, 2- to 7-day screening concentration of radon in picocuries per liter of air, which was estimated as an ordinal radon index and categorized (<2, 2-4, and >4 pCi/L) by the US Geological Survey (USGS) in 1993^14,15^ and extrapolated to county boundaries by the EPA.^16^ Primary analyses involved contemporaneously assigning these county-level, categorical radon concentrations to geocoded WHI participant addresses at baseline (1993-1998) and holding them constant throughout follow-up. Sensitivity analyses involved updating assignments when participant addresses changed during follow-up (time-varying radon concentrations) and serially replacing the primary exposure with (1) the categorized and ordinal forms of the Radon Index, (2) a county-level estimate of the 365-day mean radon concentration from the Lawrence Berkeley National Laboratory (LBNL), and (3) a US Postal Service zip code–level random-forest estimate of the 2001 to 2021 mean 2- to 7-day screening radon concentration (eMethods in Supplement 1).^3,17^

### Outcomes

We defined the first hospitalization for definite or possible acute decompensated heart failure during follow-up as the incident event. Definite decompensated heart failure indicated clear evidence of decompensation, whereas possible decompensated heart failure indicated suggestive but not definitive evidence, often due to coexisting conditions. Both definite and possible events were included to bolster sensitivity amid diagnostic uncertainty. Hospitalizations underwent a 2-step review process involving professional abstractors and physicians trained in heart failure review. Abstractors completed a standardized medical record abstraction and compiled relevant source documents. Physicians then reviewed and classified events based on symptoms, signs, diagnostic imaging, and treatment response as definite decompensated, possible decompensated, chronic stable, unlikely, or unclassifiable. Using a prespecified, mutually exclusive algorithm, ejection fractions related to the index hospitalization (from 90 days before admission through discharge) and previous 2 years were dichotomized at 50% to classify them as preserved, previously reduced but currently preserved (improved), reduced, or unknown. Preserved ejection fractions required either a current ejection fraction of 50% or greater without a previous ejection fraction of less than 50% or a current ejection fraction that was unavailable with a previous ejection fraction of 50% or greater. Improved ejection fractions required a previous ejection fraction of less than 50% and a current ejection fraction of 50% or greater. Reduced ejection fractions (study-defined systolic heart failure) required a current ejection fraction of less than 50% or, when the current ejection fraction was unavailable, a previous ejection fraction of less than 50%. Unknown ejection fractions lacked information about both current and previous measurements.^13^ Heart failure mortality, identified using the National Death Index and codes for underlying causes of death in death certificates (*International Classification of Diseases, Ninth Revision [ICD-9]* code 428.x; *International Statistical Classification of Diseases and Related Health Problems, Tenth Revision [ICD-10]* code I50.x), was physician-adjudicated for all participants from 1993 to 2010 but from 2010 to 2024, only among the medical records cohort: participants of a hormone trial, Black or African American participants, and Hispanic or Latina participants.

### Covariates

We assessed design-related, demographic, social, behavioral, clinical, and environmental characteristics of participants at baseline, including trial participation or cohort membership and hormone therapy assignment; race, ethnicity, and US census region; educational level, occupation, and neighborhood socioeconomic status; smoking, alcohol consumption, diet quality, physical activity, and sodium intake; body mass index (BMI, calculated as weight in kilograms divided by height in meters squared), blood pressure, and history of cardiometabolic conditions; and address-specific concentration of ambient fine particulate matter (<2.5 μm in diameter [PM_2.5_]) (eMethods in Supplement 1). Participants self-identified race and ethnicity using questionnaire categories modeled after the 2000 US census: race categories were Black or African American, White, and other (included American Indian or Alaska Native, Asian, Native Hawaiian or Other Pacific Islander, multiracial, and unknown or not reported) and ethnicity categories were Hispanic or Latina and not Hispanic or Latina. Race and ethnicity were included in the analysis based on previously reported differences in heart failure incidence across racial and ethnic groups.^18^

### Statistical Analysis

We estimated absolute risk of heart failure as the unadjusted cumulative incidence (cases per 1000 women at risk) and incidence rate (cases per 100 000 woman-years). We estimated the relative hazard of heart failure as a hazard ratio (HR) and 95% CI in Cox proportional hazards regression models on an attained-age time scale. Models were sequentially adjusted, beginning with design-related factors and successively adding demographic, social, behavioral, clinical, and environmental factors. To facilitate interpretation, we expressed fully adjusted HRs as equivalent increases in 2 established heart failure risk factors (systolic blood pressure and BMI). To characterize distributions of radon concentrations during follow-up, we summarized time-varying radon metrics by attained-age decade. In sensitivity analyses, we assessed whether primary results depended on additional adjustment, missing data, time scale, heart failure definition, competing mortality using Fine-Gray models, early events, radon × smoking interaction, residential mobility (temporal variation in radon concentrations following address changes), or alternative radon exposures (eMethods in Supplement 1). Data were analyzed using R, version 4.4.1 (R Project for Statistical Computing). Significance was set at 2-sided *P* <.05.

## Results

### Characteristics of Participants

Of the 43 188 included women at baseline (98% of the 44 174 WHI participants followed up for physician-adjudicated heart failure outcomes), 36%, 41%, and 23% lived in areas with indoor radon concentrations less than 2, 2 to 4, and more than 4 pCi/L, respectively (eFigure 2 in Supplement 1). A total of 75% were participants in the randomized clinical trials and 62% were in a hormone therapy arm (Table 1). Participants had a mean (SD) age of 63 (7) years and were broadly distributed across US census regions; most were college educated (59%). A total of 32% were Black or African American, 59% were White, and 9% were other race; 15% were Hispanic or Latina and 85% were not Hispanic or Latina. Homemaking was the primary occupation for 24% of participants, while ever smoking (48%) and ever consuming alcohol (86%) were common. Mean (SD) physical activity level was 11 (13) metabolic equivalent of task (MET)–hours per week, mean (SD) systolic blood pressure was 129 (18) mm Hg, and mean (SD) diastolic blood pressure was 76 (9) mm Hg; physical activity and blood pressures met guideline benchmarks in effect at baseline (≥7.5 MET-h/wk and <130/85 mm Hg, respectively).^19,20,21,22^ However, based on current guidelines, hypertension was widespread in the study population (69%), whereas hypercholesterolemia (15%), diabetes (10%), atrial fibrillation (4%), and coronary heart disease (3%) were less so. The long-term mean (SD) concentration of ambient PM_2.5_ was 12.6 (3.1) μg/m^3^, exceeding the current national standard of 9.0 μg/m^3^.^23^

**Table 1.** Characteristics of 43 188 Participants at the Screening Visit From 1993 to 1998

Characteristic	Women (N = 43 188)^a^
**Design**	
Clinical trial participants	32 411 (75)
Hormone trial arm	
Estrogen	5205 (12)
Estrogen control	5341 (12)
Estrogen plus progestin	8420 (19)
Estrogen plus progestin control	8019 (19)
Medical records cohort participants^b^	22 132 (51)
**Demographic characteristics**	
Age, mean (SD), y	63 (7)
Race	
Black or African American	13 684 (32)
White	25 693 (59)
Other^c^	3810 (9)
Ethnicity	
Hispanic or Latina	6298 (15)
Not Hispanic or Latina	36 890 (85)
US census region	
Northeast	8494 (20)
South	14 263 (33)
Midwest	9592 (22)
West	10 838 (25)
**Social characteristics**	
Educational level less than college	17 519 (41)
Occupation as homemaker	9624 (24)
Neighborhood socioeconomic status *z* score sum, mean (SD)	−2.3 (5.7)
**Behavioral characteristics**	
Smoking ever^d^	20 573 (48)
Consuming alcohol ever^e^	36 584 (86)
Healthy Eating Index score, mean (SD)^f^	63 (11)
Physical activity, mean (SD), MET-h/wk^g^	11 (13)
Sodium intake, mean (SD), mg/d	2697 (1515)
**Clinical characteristics**	
BMI, mean (SD)	29.6 (6.2)
Blood pressure, mean (SD), mm Hg	
Systolic	129 (18)
Diastolic	76 (9)
Hypertension	29 620 (69)
Hypercholesterolemia	6530 (15)
Diabetes	4124 (10)
Atrial fibrillation	1585 (4)
Coronary heart disease	1230 (3)
**Environmental characteristics**	
PM_2.5_ concentration, mean (SD), μg/m^3^	12.6 (3.1)

Abbreviations: BMI, body mass index (calculated as weight in kilograms divided by height in meters squared); MET, metabolic equivalent of task; PM_2.5_, particulate matter less than 2.5 μm in diameter.

^a^ Data are presented as the number (percentage) of participants unless otherwise indicated.

^b^ Hormone trial, Black or African American, or Hispanic or Latina participant in the second Women’s Health Initiative extension study (2010-2027).

^c^ Includes individuals self-reporting another race: American Indian or Alaska Native (140 [<1%]), Asian (500 [1%]), Native Hawaiian or Other Pacific Islander (50 [<1%]), multiracial (684 [2%]), and unknown or not reported (2436 [6%]).

^d^ Includes 4310 current smokers (10%) and 16 263 former smokers (38%).

^e^ Includes 26 519 current users (61%) and 10 065 former users (23%).

^f^ Score range, 0 to 100, with higher scores indicating better diet quality.

^g^ Total energy expenditure from recreational physical activity.

Participants in the highest EPA radon zone were predominantly in the clinical trials cohort (87%) and the medical records cohort (60%), non-Hispanic (98%) and White (75%), and residents of the Northeast (27%) or Midwest (46%) US census regions and had higher neighborhood socioeconomic status and sodium intake but lower prevalence of diabetes compared with participants in lower EPA radon zones (eTable 1 in Supplement 1). Radon metrics varied modestly across attained-age groups during follow-up (eTable 2 in Supplement 1). At ages 50 to 59 years and 90 to 99 years, the highest EPA radon zone accounted for 21% and 29% of person-years, respectively, while the respective person-year–weighted means (SDs) at those attained ages were 9.0 (2.2) and 9.5 (2.5) for the USGS Radon Index, 0.77 (0.53) pCi/L and 0.90 (0.67) pCi/L for the LBNL geometric mean radon concentration, and 1.20 (0.61) pCi/L and 1.41 (0.76) pCi/L for the random-forest mean radon concentration.

### Absolute Risk and Relative Hazards of Heart Failure

Over a mean (SD) follow-up of 16.2 (8.4) years (median, 14.8 years [IQR, 8.3-25.2 years]), there were 3446 incident (1946 [56%] definite and 1500 [44%] possible) acute decompensated heart failure events (1723 [50%] with preserved, 1152 [33%] with reduced, 554 [16%] with unknown, and 17 [<1%] with improved ejection fraction). Of the 3446 participants with incident heart failure, 259 (8%) had prevalent coronary heart disease at baseline, 498 (14%) had incident coronary heart disease before heart failure, and another 457 (13%) had concurrent (temporally and hierarchically indistinguishable) incident coronary heart disease and heart failure events. The unadjusted cumulative incidence increased with increasing radon concentration from 68 heart failure events per 1000 women at risk at less than 2 pCi/L to 79 per 1000 women at risk at 2 to 4 pCi/L and 101 per 1000 women at risk at more than 4 pCi/L. The unadjusted incidence rate also increased with increasing radon concentration from 439 heart failure events per 100 000 woman-years at less than 2 pCi/L to 488 per 100 000 woman-years at 2 to 4 pCi/L and 568 per 100 000 woman-years at more than 4 pCi/L (Figure 1 and eTable 3 in Supplement 1). By age 90 years, the cumulative incidence reached 9.0% at less than 2 pCi/L, 10.0% at 2 to 4 pCi/L, and 11.9% at more than 4 pCi/L.

**Figure 1.**
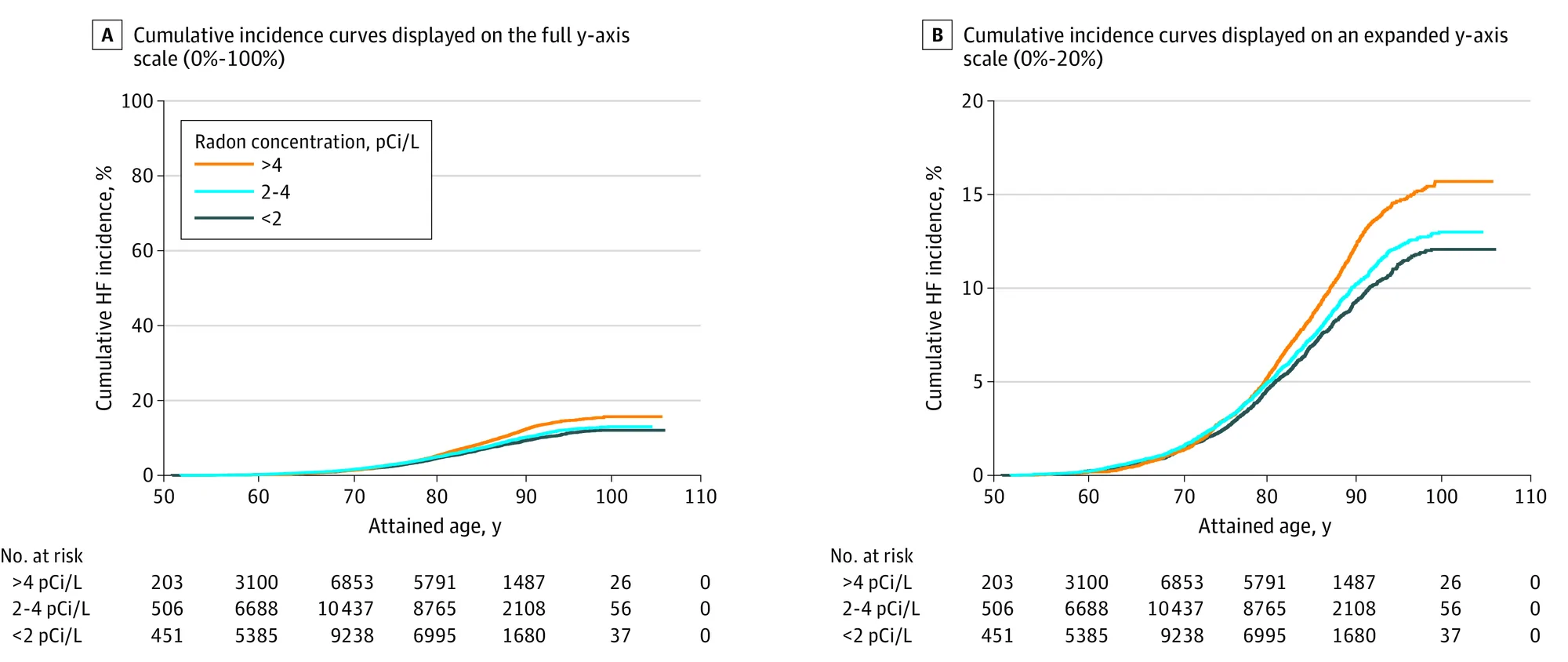
Kaplan-Meier Curves of Cumulative Incidence and Incidence Rate of Heart Failure by US Environmental Protection Agency (EPA) Radon Concentration, 1993-2024 Cumulative incidence of heart failure among women at risk at attained ages of 50 to 110 years is shown across EPA radon concentrations (<2, 2-4, and >4 picocuries [pCi]/L) over an attained-age time scale, estimated using nonparametric methods with no adjustment for covariates. The inset plots the same data on an expanded y-axis.

Compared with exposure to radon concentrations less than 2 pCi/L, the unadjusted HR for heart failure at 2 to 4 pCi/L was 1.09 (95% CI, 1.00-1.19) and at more than 4 pCi/L was 1.16 (95% CI, 1.05-1.27) (*P* = .01), changing little with cumulative adjustment for covariates; the fully adjusted HR was 1.19 (95% CI, 1.07-1.33) at more than 4 pCi/L vs less than 2 pCi/L (Table 2). The HR at more than 4 pCi/L approximated that of an increase of 16 mm Hg in systolic blood pressure and of 4.8 in BMI.

**Table 2.** Relative Hazard of Heart Failure by US EPA Radon Concentration From 1993 to 2025

Cumulative analysis	HR (95% CI)		*P* value^b^
	2-4 pCi/L^a^	>4 pCi/L^a^	
Unadjusted	1.09 (1.00-1.19)	1.16 (1.05-1.27)	.01
Plus design^c^	1.09 (1.00-1.19)	1.14 (1.03-1.25)	.03
Plus demographic factors^d^	1.09 (0.99-1.19)	1.15 (1.04-1.29)	.03
Plus social factors^e^	1.11 (1.01-1.21)	1.18 (1.05-1.31)	.01
Plus behavioral factors^f^	1.09 (1.00-1.19)	1.18 (1.06-1.32)	.009
Plus clinical factors^g^	1.09 (0.99-1.19)	1.19 (1.06-1.33)	.009
Plus environmental factors^h^	1.09 (0.99-1.20)	1.19 (1.07-1.33)	.008

Abbreviations: EPA, Environmental Protection Agency; HR, hazard ratio; pCi, picocuries.

^a^ The reference group is less than 2 pCi/L.

^b^ *P* values were calculated using likelihood ratio tests.

^c^ Adjusted for clinical trial participation, medical records cohort membership, and hormone therapy trial arm assignment.

^d^ Additionally adjusted for race, ethnicity, and US census region.

^e^ Additionally adjusted for educational level, occupation, and neighborhood socioeconomic status.

^f^ Additionally adjusted for smoking status, alcohol consumption, diet quality, physical activity, and sodium intake.

^g^ Additionally adjusted for body mass index, systolic and diastolic blood pressure, and history of hypercholesterolemia, diabetes, atrial fibrillation, hypertension, and coronary heart disease.

^h^ Additionally adjusted for the geocoded, address-specific 3-year mean concentration of ambient fine particulate matter less than 2.5 μm in diameter.

### Sensitivity Analyses

The aforementioned patterns were generally consistent in sensitivity analyses (Figure 2 and eFigure 3 in Supplement 1), changing negligibly with adjustment for center or comorbidities, multiple imputation, time scale, exclusion of early events, and substitution of USGS radon zones for EPA radon zones. HRs were somewhat higher for heart failure with reduced vs preserved ejection fraction (*P* = .07 for interaction), with HRs of 1.24 (95% CI, 1.04-1.49) for heart failure with reduced ejection fraction and 1.37 (95% CI, 1.12-1.69) for heart failure mortality at radon concentrations greater than 4 pCi/L vs less than 2 pCi/L, although the HRs were not significantly different for concentrations of 2 to 4 pCi/L vs less than 2 pCi/L. Other analyses yielded higher HRs after accounting for the competing risk of death, among ever vs never smokers (*P* = .02 for interaction), and for baseline vs time-varying radon concentrations. Moreover, HRs increased per unit increase in the USGS Radon Index (HR, 1.03; 95% CI, 1.01-1.05), per 1-pCi/L increase in the LBNL geometric mean radon concentration (HR, 1.10; 95% CI, 1.02-1.19), and per 1-pCi/L increase in the random-forest mean radon concentration (HR, 1.11; 95% CI, 1.05-1.18).

**Figure 2.**
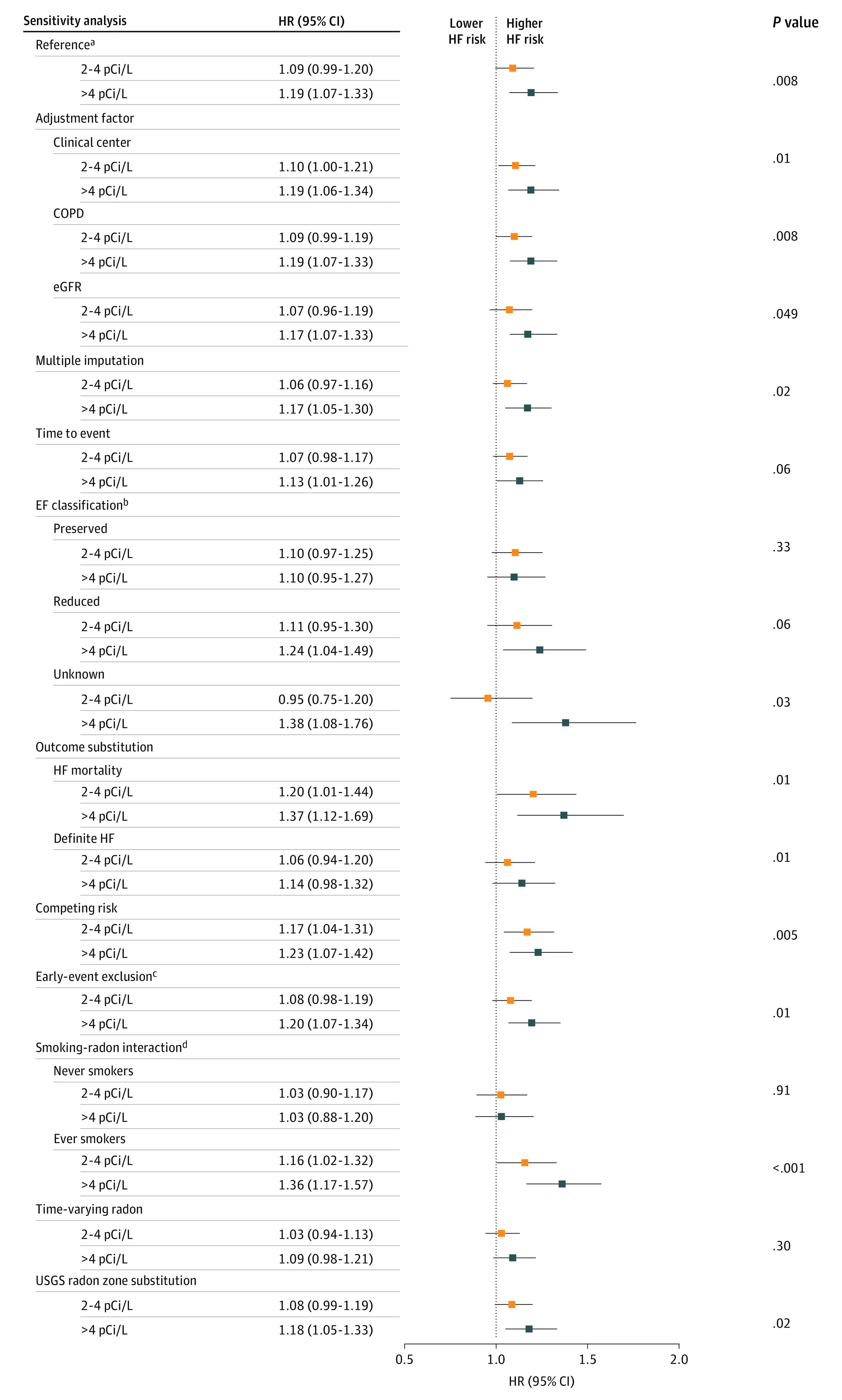
Dot-and-Whisker Plot for Sensitivity Analyses of the Fully Adjusted Relative Hazard of Heart Failure (HF) by US Environmental Protection Agency (EPA) Radon Concentration, 1993-2024 Hazard ratios (HRs) with 95% CIs for heart failure are from sensitivity analyses comparing concentrations of more than 4 picocuries (pCi)/L and 2 to 4 pCi/L with less than 2 pCi/L (reference category). *P* values are from the likelihood ratio test. COPD indicates chronic obstructive pulmonary disease; EF, ejection fraction; eGFR, estimated glomerular filtration rate (based on an analysis restricted to participants with baseline creatinine measurements); USGS, US Geological Survey. ^a^Fully adjusted model of the association between EPA radon concentration and HF from Table 2. ^b^Global *P* value for EPA radon concentration × EF classification is .07. ^c^Excludes HF events within the first 3 years of follow-up. ^d^Global *P* value for EPA radon concentration × smoking interaction is .02.

## Discussion

In this large, well-characterized cohort of postmenopausal women in the US followed up for more than 16 years, we observed positive associations between indoor radon concentrations and incident, physician-adjudicated heart failure. The HRs were greater for heart failure with reduced ejection fraction, for heart failure mortality, after adjustment for competing risk of death, among ever smokers, and with radon concentrations at baseline, but patterns were otherwise consistent across sensitivity analyses. The associations with heart failure at a radon concentration more than 4 pCi/L also approximated that of an increase of 16 mm Hg in systolic blood pressure and of 4.8 in BMI, which are 2 established risk factors for heart failure (eMethods in Supplement 1).^24,25^ Collectively, these patterns highlight indoor radon concentration as a potentially important environmental factor associated with incident heart failure among postmenopausal women in the US.

The potential importance of these findings aside, radon policy in the US is currently driven by an EPA action level of 4 pCi/L based solely on the estimated risk of lung cancer.^26,27^ However, concern about relationships between radon and relatively common, societally burdensome forms of cardiovascular disease and its sequelae^28,29^ emerged nearly 30 years ago in occupational cohort studies of fluorspar miners.^30,31^ More recently, residential radon concentrations at roughly half the EPA action level and radon-related ambient-particle radioactivity also have been associated with heart failure risk factors,^5,6,7,8^ related cardiovascular outcomes,^9,10,11^ and mortality among patients with heart failure.^12^ Conversely, Turner et al^32^ found no association between county-level residential radon concentrations and heart disease mortality in the American Cancer Society cohort, whose participants were generally younger at baseline than those in the WHI cohort, whereas Henyoh et al^33^ found no significant meta-analytically pooled associations between occupational radon exposure among miners and circulatory or ischemic heart disease mortality.^33^ The present study thereby extends this mixed literature by evaluating incident physician-adjudicated heart failure, its ejection fraction phenotypes, and heart failure mortality.

The association between increased radon exposure and incident heart failure is biologically plausible because exposure to radon-related ionizing radiation may damage endothelium, cardiomyocytes, or leukocytes, mutating their DNA and thereby inducing necrosis, apoptosis, and/or clonal hematopoiesis. The latter pathophysiologic process is jointly associated with indoor radon concentrations and heart failure in postmenopausal women^34,35,36^ and experimentally supported by murine models.^37,38^ Tissue-damaging α-particle radioactivity may be related to the chain of cardiomyocyte cell death, sterile inflammation, angiogenesis, fibrosis, and altered contractility that more often underlies the systolic dysfunction characteristic of heart failure with reduced ejection fraction than the metabolic risk–induced inflammation typically underlying the diastolic dysfunction characteristic of heart failure with preserved ejection fraction.^39^ Such distinctions may help explain why higher radon concentrations in the present study were associated with a greater risk of heart failure with reduced than preserved ejection fraction. Adjustment for coronary heart disease, modeled as a time-varying covariate, also failed to attenuate the radon–heart failure associations either overall or by subtype—a result suggesting that the observations are unlikely to be fully explained by preceding coronary syndromes despite a report that α-particle radioactivity was 4 times higher in autopsied coronary arteries of decedents with ischemic heart disease vs other causes of death.^40^ Excluding early heart failure events yielded results similar to those of the primary analysis, reducing concern about the putative influence of preexisting disease, yet these were not formal mediation analyses; thus, the findings should be interpreted as supportive rather than definitive evidence of a partially noncoronary mechanism of disease. In this study, smoking status also modified the association between radon exposure and heart failure, with positive associations observed among ever smokers but not never smokers. This pattern mimics the established, radon-related modification of smoking effects on lung cancer^4^ and may be compatible with amplification of the aforementioned oxidative and inflammatory processes.

### Limitations

This study has several methodologic limitations, with exposure assessment arguably warranting the greatest consideration. Although outcomes and covariates were measured at the participant level, exposures were estimated by linking participants’ residential addresses to area-level radon concentrations. This combination of participant-level outcomes and covariates with area-level exposures constitutes a partially ecologic study design.^41^ Such designs prevent characterizing variation in exposure among participants within the same area and its associations with their outcomes and covariates, potentially introducing exposure misclassification and residual spatial confounding. The direction of any resulting bias is uncertain because attenuation toward the null, as occurs with classic measurement error in simple linear regression, is not guaranteed in other contexts.^42^

Although simultaneous reliance on participant-level outcome and covariate data limits the magnitude of the aforementioned bias relative to fully ecologic designs, EPA radon zones—the primary exposure used in this study—did not capture within-zone or seasonal variation of indoor radon concentrations among participant homes^16^ (or prior home mitigation, despite its low [approximately 2%] prevalence among the highest-radon homes^43^). The associations with higher risk of heart failure observed with baseline than time-varying radon concentrations may thereby reflect the proximity of the WHI baseline time point to the measurement period underlying the EPA, USGS, and LBNL exposure metrics; differences in the validity of earlier-life vs late-life approximations of radon exposure; and/or secular increases in the potential for exposure overestimation. EPA radon zones are based on an oversample of high-radon homes in the State Residential Radon Survey^44^ and thus may overestimate true radon exposures. By design, the EPA zones nevertheless reflected population-level radon potential and aligned with measured concentrations in the original validation, wherein 72% of county-level means fell within anticipated EPA zones and the remainder differed by no more than 1 zone.^45^ The State Residential Radon Survey indoor radon concentrations on which EPA zones are based also agree with contemporary radon test results, suggesting that annual mean indoor radon concentrations have remained relatively stable over decades.^3,46^ Furthermore, the USGS Radon Index, EPA zones, and LBNL mean are all moderately to strongly correlated with the higher-resolution US Postal Service zip code–level estimate of the mean screening concentration from the random-forest model: *r^2^* = 0.60 for Radon Index, *r^2^* = 0.64 for EPA zones, and *r^2^* = 0.69 for the LBNL mean. Sensitivity analyses substituting these alternative exposure metrics yielded consistently positive radon–heart failure associations. However, these metrics may share a common, currently unrecognized source of exposure misclassification; thus, the consistency of the findings does not eliminate this concern.

The observed associations were not fully explained by measured demographic, neighborhood socioeconomic, or environmental factors, yet substantial confounding also appears to have been unlikely given the broad adjustment for design-related, demographic, social, behavioral, clinical, and environmental factors and extensive sensitivity analyses. In particular, given the strong regional distribution of indoor radon concentrations (eFigure 2 in Supplement 1), we adjusted for US census region and examined center-level random effects, thereby reducing the likelihood that the observed associations were driven by unmeasured geographic factors. Nevertheless, residual confounding cannot be excluded, whether by unmeasured contextual characteristics or, in the smoking-stratified analyses, by aspects of smoking history not captured by the ever-never classification, including smoking intensity, duration, and time since cessation. Potential mediation also remains unresolved. Inclusion of time-varying coronary heart disease in the primary model did not materially alter the estimates, but the study was not designed to formally assess mediation and therefore cannot exclude coronary heart disease as a potential mediator. Likewise, we adjusted for baseline atrial fibrillation but lacked data on incident atrial fibrillation and other, potentially mediating arrhythmias. Heart failure mortality was based on the *ICD-9*– and *ICD-10*–coded underlying cause of death and was not uniformly adjudicated; therefore, some cause-of-death misclassification was possible. Generalizability of the associations also remains limited to postmenopausal women in the sixth or later decades of life, although by design, the study population is more racially and ethnically diverse than is typical in investigations drawing from the parent WHI cohort. Additional studies that include men, younger adults, and other races and ethnicities are needed.

## Conclusions

In this large, prospective cohort study of US postmenopausal women, we found a positive association between indoor radon concentrations and incident, physician-adjudicated heart failure. Future studies of carefully validated indoor radon concentrations and 1-time interventions that can cost-effectively lower them (eg, home mitigation) among more diverse populations could further clarify these associations. Evidence emerging from such studies that reflects the ubiquity of radon gas in US homes and reinforces its role as a modifiable determinant of incident heart failure, myocardial infarction, stroke, or their risk factors could inform future reconsideration of extant radon policies, the dated cost-benefit analyses of lung cancer on which they are based, and if appropriate, potentially synergistic cardiovascular disease prevention strategies. Although the cardiovascular health benefits of radon mitigation for all household members remain unknown, they are conceivable, perhaps as they once were for smoking cessation long before cigarette smoking and environmental tobacco smoke were recognized as risk factors for nonpulmonary disease. By analogy, the benefits of radon mitigation may eventually extend beyond its efficient structural prevention of lung cancer.

## Notes

Supplement 1. eMethods. eTable 1. Characteristics of 43 188 Participants at the Screening Visit (1993-1998), by EPA Radon Zone eTable 2. Distribution of Radon Concentrations by Attained-Age Decade During Follow-Up eTable 3. Cumulative Incidence, Incidence Rates, and Unadjusted Hazard Ratios for Incident Heart Failure by EPA Radon Zone eFigure 1. Flow Diagram of Participant Eligibility, Exclusion, and Follow-Up (Women’s Health Initiative, 1993-2024) eFigure 2. Distribution of Participants at the Baseline Visit, by US Environmental Protection Agency Radon Concentration and US Census Region (Women’s Health Initiative, 1993-1998) eFigure 3. Sensitivity of the Fully Adjusted Relative Hazard for Heart Failure Subtypes by US EPA Radon Concentration (1993-2024) eReferences

Supplement 2.
Data Sharing Statement
