# Supplementary material for: Indoor Radon and Incident Heart Failure Among Postmenopausal Women
Source: JAMA Netw Open. 2026 Nov 5;9(11):e2642391. doi: 10.1001/jamanetworkopen.2026.42391 (PMC13741163; doi:10.1001/jamanetworkopen.2026.42391)
Supplement: Supplementary file 1 [file jamanetwopen-e2642391-s001.pdf]

Supplementary Online Content

Chen S, Shi C, Collins JM, et al. Indoor radon and incident heart failure among postmenopausal women. *JAMA Netw Open*. 2026;9(11):e2642391. doi:10.1001/jamanetworkopen.2026.42391

eMethods

eTable 1. Characteristics of 43 188 Participants at the Screening Visit (1993-1998), by EPA Radon Zone

eTable 2. Distribution of Radon Concentrations by Attained-Age Decade During Follow-Up

eTable 3. Cumulative Incidence, Incidence Rates, and Unadjusted Hazard Ratios for Incident Heart Failure by EPA Radon Zone

eFigure 1. Flow Diagram of Participant Eligibility, Exclusion, and Follow-Up (Women's Health Initiative, 1993-2024)

eFigure 2. Distribution of Participants at the Baseline Visit, by US Environmental Protection Agency Radon Concentration and US Census Region (Women's Health Initiative, 1993-1998)

eFigure 3. Sensitivity of the Fully Adjusted Relative Hazard for Heart Failure Subtypes by US EPA Radon Concentration (1993-2024)

eReferences

This supplementary material has been provided by the authors to give readers additional information about their work.

© 2026 Chen S et al. JAMA Network Open.

eMethods

Study Population

The Women's Health Initiative (WHI) is a large, prospective cohort study of risk factors for major causes of morbidity and mortality in postmenopausal women in the United States. Between 1993 and 1998, 161,808 women aged 50 to 79 years were recruited at 40 clinical centers nationwide and enrolled into either the Observational Study (OS; n = 93,676) or one or more of three Clinical Trials (CT; n = 68,132) evaluating hormone therapy, dietary modification, and calcium/vitamin D supplementation. All participants provided written informed consent.[1]

At baseline, participants completed a standardized screening visit, which included questionnaires on demographics, lifestyle, diet, and medical history; physical measurements; and collection of blood specimens. CT participants were followed with annual clinic visits for up to 9 years, and OS participants with a Year 3 clinic visit and mailed questionnaires thereafter, for up to 8 years. Study protocols and procedures have been described in detail previously.[2]

Following the end of the main study, continued follow-up was conducted through two extension studies (Extension 1, 2005–2010; Extension 2, 2010–2027) and the WHI Medical Records Cohort (MRC, 2010–2027),[3] which enabled long-term outcome ascertainment from medical records. Outcomes were identified by participant self-report, verified through medical record abstraction, and centrally adjudicated using standardized protocols.

Exposures

The U.S. Environmental Protection Agency (EPA) classification of estimated indoor radon concentrations served as the primary exposure metric. Areal classification was based on a continuous radon index developed by the U.S. Geological Survey (USGS), which scored and summed 2-7 day, indoor radon concentrations plus architectural, geologic, edaphic, and aerial factors into a unitless index (range 3–17), then trichotomized it into geologic radon potential concentrations: <2, 2-4, and >4 picoCuries/liter (pCi/L). U.S. EPA then extrapolated them to county boundaries.[4,5,6] In parallel, the E.O. Lawrence Berkeley National Laboratory (LBL) produced continuous, county-level estimates using hierarchical Bayesian models that combined the 2-7 day and 1 year indoor radon concentrations with architectural and geologic factors (range: 0.12–5.06).[7,8] The above legacy metrics were augmented with a more recently published U.S. Postal Service Zone Improvement Plan (ZIP) code-level estimate of the 2001-2021 mean, 2-7 day screening concentrations of radon from a random forest model (range: 0.34–6.65).[9]

Covariates

Baseline covariates were grouped into six domains: design-related, demographic, social, behavioral, clinical, and environmental. Design-related covariates included clinical trial participation, Medical Records Cohort membership, and hormone therapy trial arm assignment (estrogen, estrogen control, estrogen plus progestin, estrogen plus progestin control, or not randomized). Demographic characteristics included race (White, Black/African American, or other), ethnicity (Hispanic/Latina or not), and U.S. Census region (Northeast, South, Midwest, or West). Social characteristics included education (<college or ≥college), occupation (homemaker or not), and neighborhood socioeconomic status, a census tract–based summary z-score.[10] Behavioral characteristics included smoking status (ever/never), alcohol consumption (ever/never), diet quality as assessed by the 2015 Healthy Eating Index (calculated from a validated food-frequency questionnaire), physical activity measured in metabolic equivalent (MET)–hours per week,[11] and sodium intake (mg/day). Clinical characteristics included body mass index (weight in kg/ height in m^2), systolic and diastolic blood pressure (mm Hg), and histories of hypercholesterolemia, diabetes mellitus, atrial fibrillation, hypertension, and time-varying coronary heart disease (CHD). CHD was defined at baseline as a self-reported history of myocardial infarction, coronary artery bypass grafting, or angioplasty (no/yes). Among participants without CHD at baseline, CHD status was updated from no to yes on the date of the first incident CHD event during follow-up and remained yes thereafter. Other clinical histories were defined by self-report, relevant medication use, or blood pressure measurements. Finally, environmental characteristics included the geocoded participant address-specific, three-year mean concentration of ambient fine particulate matter ($PM_{2.5}$). Missingness across most covariates was low (<5%), with the highest proportions observed for physical activity (6.6%), homemaker (8%), and the baseline measure of eGFR used in sensitivity analysis (54%, because creatinine was only available in selected WHI subcohorts).

Statistical Analysis

Given low and non-differential covariate missingness with respect to radon concentrations and incident heart failure, we based primary results on a complete-case analysis but assessed their robustness in sensitivity analyses

© 2026 Chen S et al. JAMA Network Open.

(see below). We estimated the relative hazards of heart failure across U.S. EPA radon concentrations as a hazard ratio (95% confidence interval) using Cox proportional hazards regression models[12] with attained age as the time scale.[13] Models progressively incorporated covariates from the six prespecified domains described above. We implemented all primary models using the *survival* package in R.[14]

We evaluated the proportional hazards assumption using scaled Schoenfeld residuals, which assess whether residuals are systematically correlated with attained age.[15] We performed individual tests of each covariate in the model, including radon concentration, and a global test jointly assessing proportionality across all covariates in the model. We implemented both tests using the *cox.zph()* function of the *survival* package in R. The global test indicated significant violation of proportional hazards ($P < .001$), consistent with visual evidence of crossing cumulative hazard functions across radon concentrations. To address the non-proportionality, we stratified Cox models by covariates with evidence of non-proportionality in individual Schoenfeld tests[16] (hormone therapy trial arm, Medical Records Cohort membership, race, smoking status, history of hypertension, and history of diabetes), implemented using the *strata()* function of the *survival* package in R. After stratification, hazards were proportional, with non-significant, covariate-specific ($P = .64$) and global ($P = .24$) tests.

To facilitate interpretation of hazard ratios from the fully adjusted Cox models, we expressed them in terms of equivalent increases in two established risk factors for heart failure[17,18] by dividing the estimated coefficient for radon concentrations >4 pCi/L (β=0.176) by that for a 1 mm Hg increase in systolic blood pressure (β=0.011) and alternatively, a 1 kg/m^2 increase in body mass index (β=0.037). The resulting increments correspond to the same fitted log-hazard contrast, conditional on the other model covariates, and do not imply causal, mechanistic, or interventional equivalence.

Sensitivity Analyses

We conducted several sensitivity analyses to evaluate robustness of findings. (1) To account for potential clustering by recruitment site, we refitted models including a frailty term for WHI clinical center while retaining other covariates as fixed effects.[22] To address the possibility of confounding by pulmonary or renal comorbidities at baseline, we added self-reported history of chronic obstructive pulmonary disease (emphysema or chronic bronchitis) and the glomerular filtration rate (mL/min/1.73 m^2) estimated from serum creatinine using the 2021 Chronic Kidney Disease Epidemiology Collaboration equation[23] to the multivariable-adjusted models of heart failure and as described under (4), below, its subtypes. (2) We addressed missing covariate data using multiple imputation by chained equations.[24] We combined estimates from analyses of five imputed datasets using Rubin's rules[25] implemented with the *pool()* function of the *mice* package in R. (3) We repeated analyses using time since enrollment, rather than attained age, as the time scale. (4) We substituted alternative adjudicated outcomes, including heart failure mortality, definite decompensated heart failure only, and three mutually exclusive heart failure subtypes: with preserved, reduced, or unknown ejection fraction. We applied a joint Cox proportional hazards model stratified by subtype[26] to allow for subtype-specific baseline hazards while estimating a common radon concentration-heart failure association across subtypes. To evaluate heterogeneity, we added a radon-heart failure subtype interaction term to the model and conducted a global Wald test of significance. (5) To account for the competing risk of death, we also estimated the subdistribution hazard ratio using Fine–Gray models[27] implemented with the *prep.comp.risk* and *comp.risk* functions of the *timereg* package in R.[28] The functions allowed for (left truncation of) the attained age time scale, thereby accommodating differences in baseline age at enrollment. We defined the competing event as all-cause mortality (including heart failure caused deaths) during follow-up through February 17, 2024. We adjusted for the same covariates included in the primary Cox model, except that CHD was included as a baseline instead of time-varying covariate because time-updated CHD could not be accommodated in the Fine–Gray model. We assessed proportionality of subdistribution hazards by visual inspection of both cumulative incidence functions and time-varying coefficient plots, $\beta(t)$, knowing that when proportionality holds, the radon concentration-specific cumulative incidence functions do not cross and the estimated regression coefficient $\beta(t)$ remains approximately constant over time.[15] Both sets of plots showed no meaningful departures from proportionality herein. (6) To protect against reverse causality, we excluded early events (heart failure within the first three years of follow-up). (7) To evaluate whether smoking status modified the radon-heart failure association, we added two cross-product terms between the three-category EPA radon zone variable and smoking status (ever vs never) to the fully adjusted Cox model. (8) Finally, we conducted two sets of radon sensitivity analyses. First, to account for residential mobility, we updated the assigned primary radon concentration when a participant's geocoded residential address changed, using residential histories available from enrollment through 2013. Second, we substituted alternative radon metrics, including the USGS radon concentration, USGS radon index, LBL geometric mean radon concentration, and ZIP code–level radon estimate derived from a national random forest model. We modeled the USGS radon index, the LBL geometric mean, and the ZIP code-level estimate both linearly and using restricted cubic splines with three knots to evaluate potential non-linearity, implementing the spline using the *rcs()* function of the *rms* package in R.[29] For all three exposures,

© 2026 Chen S et al. JAMA Network Open.

model comparison (i.e., Akaike and Bayesian information criteria) favored linear specifications over spline functions. Unless otherwise specified, sensitivity analyses used the fully adjusted Cox proportional hazards model with attained age as the time scale.

Power

With a total sample size of 43,188 participants across radon concentrations <2, 2-4, and > 4 pCi/L, the study had >80% power to detect minimum hazard ratios of 1.12 and 1.13 when comparing the two highest to the lowest concentration, assuming a two-sided $\alpha = 0.05$.

© 2026 Chen S et al. JAMA Network Open.

eTable 1. Characteristics of 43,188 Participants at the Screening Visit (1993-1998), by EPA Radon Zone

Characteristic		Mean (SD) or n (%)		
		>4 pCi/L	2-4 pCi/L	<2 pCi/L
**Design**	Clinical Trials participant	8,617 (87%)	13,108 (74%)	10,686 (69%)
	Hormone Trial arm			
	Estrogen	1,408 (14%)	2,064 (12%)	1,733 (11%)
	Estrogen control	1,487 (15%)	2,068 (12%)	1,786 (12%)
	Estrogen + progestin	2,542 (26%)	3,243 (18%)	2,635 (17%)
	Estrogen + progestin control	2,392 (24%)	3,112 (18%)	2,515 (16%)
	Medical Records Cohort[a] participant	5,929 (60%)	9,006 (51%)	7,197 (46%)
**Demographic**	Age (yr)	63.0 (7)	62.6 (7)	62.2 (7)
	Race			
	Black/African American	2,209 (22%)	6,206 (35%)	5,268 (34%)
	White	7,440 (75%)	9,808 (56%)	8,445 (54%)
	Other[b]	291 (3%)	1,668 (9%)	1,850 (12%)
	Ethnicity Hispanic/Latina	243 (2%)	2665 (15%)	3,389 (22%)
	U.S. Census Region			
	Northeast	2,709 (27%)	2,884 (16%)	2,901 (19%)
	South	2,315 (23%)	4,697 (27%)	7,251 (47%)
	Midwest	4,574 (46%)	3,959 (22%)	1,058 (7%)
	West	342 (3%)	6,142 (35%)	4,353 (28%)
**Social**	Education < college	4,097 (41%)	6,961 (39%)	6,459 (42%)
	Occupation = homemaker	2,317 (23%)	3,947 (22%)	3,359 (22%)
	Neighborhood socioeconomic status (z score sum)	−1.0 (5)	−2.1 (6)	−3.4 (6)
**Behavioral**	Smoke Ever[c]	4,712 (47%)	8,601 (49%)	7,260 (47%)
	Alcohol Ever[d]	8,642 (87%)	15,067 (85%)	12,873 (83%)
	Healthy Eating Index	63.8 (11)	63.0 (11)	63.3 (11)
	Physical activity[e] (MET-h/wk)	10.6 (13)	10.7 (14)	10.5 (13)
	Sodium intake (mg/day)	2,752.2 (1,352)	2,725.3(1,604)	2,629.7 (1,507)
**Clinical**	Body mass index (kg/m^2)	29.3 (6)	29.6 (6)	29.6 (6)
	Systolic pressure (mm Hg)	128.6 (18)	129.2 (18)	129.5 (18)
	Diastolic pressure (mm Hg)	76.1 (9)	76.2 (9)	77.0 (9)
	Hypertension	6,679 (67%)	12,111 (69%)	10,829 (70%)
	Hypercholesterolemia	1,469 (15%)	2,539 (14%)	2,522 (16%)
	Diabetes	788 (8%)	1,672 (10%)	1,664 (11%)
	Atrial fibrillation	355 (4%)	652 (4%)	578 (4%)
	Coronary Heart Disease	302 (3%)	514 (3%)	414 (3%)
**Environmental**	$PM_{2.5}$ (μg/m^3)	12.9 (3)	12.2 (4)	12.9 (3)

MET indicates metabolic equivalent of task; n, number of participants; pCi/L, picocuries per liter; $PM_{2.5}$, particulate matter with an aerodynamic diameter of 2.5 μm or less; and SD, standard deviation. [a] Hormone Trial, Black/African American, or Hispanic/Latina participant in Extension 2. [b] Includes individuals self-reporting another race: Native Hawaiian or Other Pacific Islander (0.1%), American Indian or Alaska Native (0.3%), Asian (1.2%), more than one race (1.6%), and unknown or not reported (5.8%).[c] Includes 4,310 (10%) current and 16,263 (38%) former smokers.[d] Includes 26,520 (62%) current and 10,065 (24%) former users. [e] Total energy expenditure from recreational physical activity.

© 2026 Chen S et al. JAMA Network Open.

eTable 2. Distribution of Radon Concentrations by Attained-Age Decade During Follow-up

Radon Concentration Metrics		Person-years (%) or Person-Year-Weighted Mean (SD)						
		Overall	50–59	60–69	70–79	80–89	90–99	100–109
EPA Radon Zone[a]	>4 pCi/L	175,584 (25%)	15,959 (21%)	51,595 (24%)	67,183 (26%)	35,499 (28%)	5,305 (29%)	43 (23%)
	2-4 pCi/L	279,300 (40%)	30,427 (40%)	87,824 (40%)	103,291 (40%)	50,639 (40%)	7,042 (39%)	75 (41%)
	<2 pCi/L	245,240 (35%)	30,056 (39%)	78,514 (36%)	89,093 (34%)	41,684 (33%)	5,827 (32%)	66 (36%)
USGS Radon Index[b]	unitless	9.3 (2.4)	9.0 (2.2)	9.3 (2.3)	9.4 (2.4)	9.5 (2.5)	9.5 (2.5)	9.3 (2.3)
LBL Mean[b]	pCi/L	0.85 (0.62)	0.77 (0.53)	0.83 (0.60)	0.86 (0.63)	0.89 (0.66)	0.90 (0.67)	0.75 (0.52)
Random Forest Mean[b]	pCi/L	1.33 (0.71)	1.20 (0.61)	1.30 (0.69)	1.35 (0.72)	1.40 (0.74)	1.41 (0.76)	1.25 (0.64)

[a] Values are person-years (%) for categorical metrics. [b] Values are person-year-weighted means (standard deviations) for continuous metrics.

© 2026 Chen S et al. JAMA Network Open.

eTable 3. Cumulative Incidence, Incidence Rates, and Unadjusted Hazard Ratios for Incident Heart Failure by EPA Radon Zone.

Radon Concentrations	Cumulative Incidence[a]	Incidence Rate[b]	Hazard Ratio (95% CI)[c]
<2 pCi/L	68	439	1.00
2-4 pCi/L	79	488	1.09 (1.00-1.19)
>4 pCi/L	101	568	1.16 (1.05-1.27)

pCi/L = picoCuries per liter. [a] Per 10^3 women. [b] Per 10^5 woman-years. [c] P = .01.

© 2026 Chen S et al. JAMA Network Open.

eFigure 1. Flow Diagram of Participant Eligibility, Exclusion, and Follow-up (Women's Health Initiative, 1993-2024).

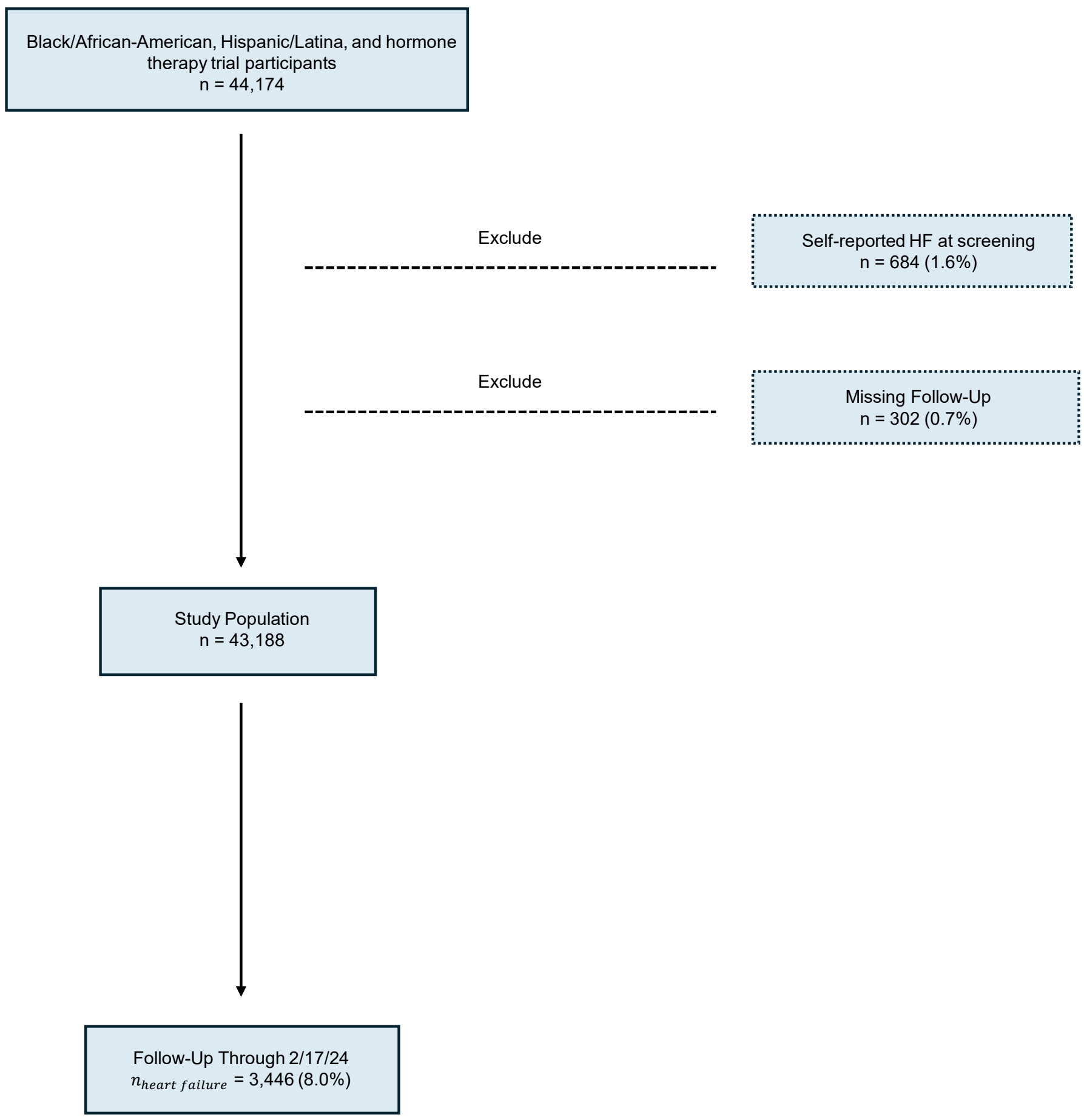

eFigure 1. Flow Diagram of Participant Eligibility, Exclusion, and Follow-up (Women's Health Initiative, 1993-2024). Of 44,174 women with adjudicated outcomes (all black/African American, Hispanic/Latina, and hormone therapy trial participants), we excluded 684 with self-reported heart failure at screening and 302 with missing follow-up data. The resulting eligible cohort included 43,188 women, among whom 3,446 hospitalizations for incident, definite or possible, acute decompensated heart failure (8.0%) were ascertained during follow-up.

© 2026 Chen S et al. JAMA Network Open.

eFigure 2. Distribution of Participants at the Baseline Visit, by U.S. Environmental Protection Agency Radon Concentration and U.S. Census Region (Women's Health Initiative, 1993–1998).

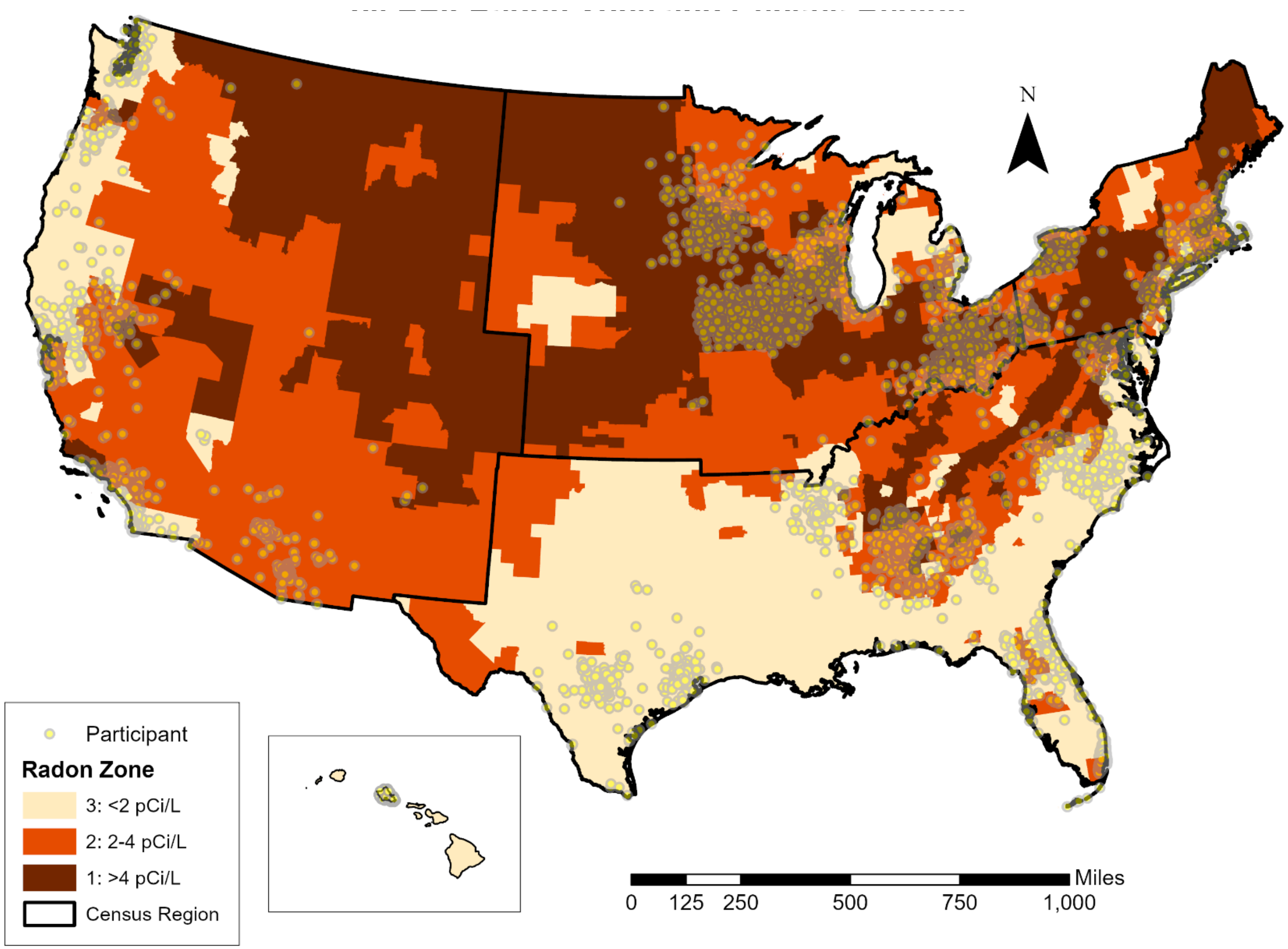

eFigure 2. Distribution of Participants at the Baseline Visit, by U.S. Environmental Protection Agency Radon Concentration and U.S. Census Region (Women's Health Initiative, 1993–1998). Of the 43,188 participants at baseline, 15,563 (36%), 17,683 (41%), and 9,940 (23%) lived in areas with indoor radon concentrations <2, 2-4, and >4 pCi/L.

© 2026 Chen S et al. JAMA Network Open.

eFigure 3. Sensitivity of the Fully Adjusted Relative Hazard[a] for Heart Failure Subtypes by U.S. EPA Radon Concentration (1993–2024).

Sensitivity to	EF Classification	2–4 pCi/L[b]	>4 pCi/L[b]	P[LRT]
Referent:[c]	preserved	1.10 (0.97, 1.25)	1.10 (0.95, 1.27)	.33
	reduced	1.11 (0.95, 1.30)	1.24 (1.04, 1.49)	.06
	unknown	0.95 (0.75, 1.20)	1.38 (1.08, 1.76)	.03
Adjustment for COPD[d]	preserved	1.01 (0.99, 1.26)	1.11 (0.96, 1.29)	.28
	reduced	1.09 (0.92, 1.27)	1.22 (1.02, 1.47)	.09
	unknown	0.94 (0.75, 1.19)	1.38 (1.08, 1.76)	.02
Adjustment for eGFR[e]	preserved	1.07 (0.93, 1.24)	1.06 (0.93, 1.25)	.65
	reduced	1.11 (0.93, 1.34)	1.28 (1.04, 1.58)	.11
	unknown	0.92 (0.70, 1.21)	1.37 (1.03, 1.83)	.04

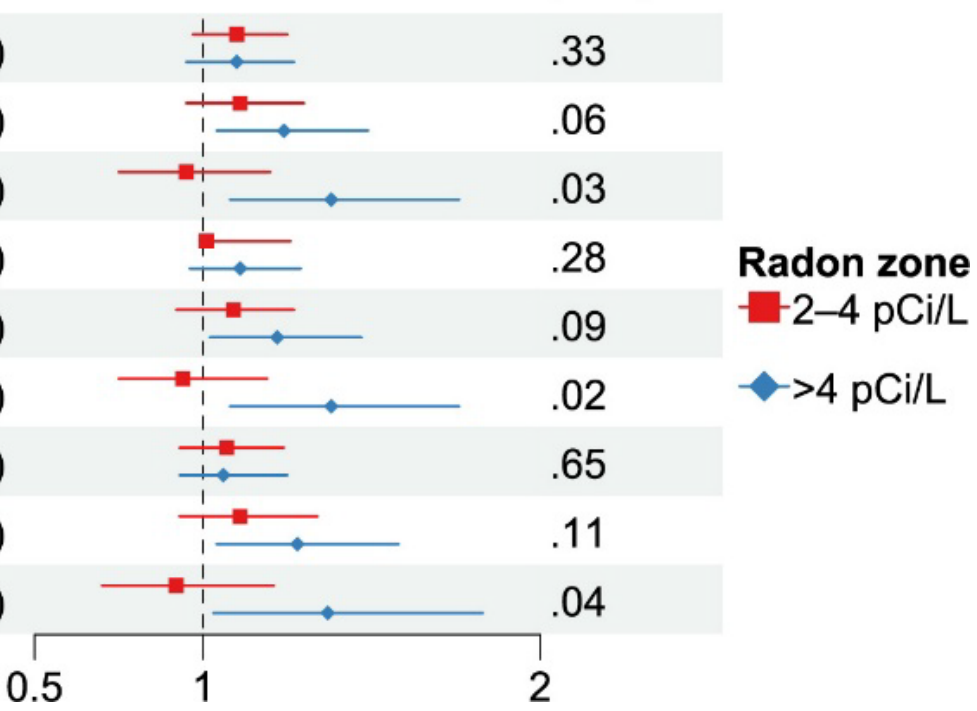

eFigure 3. Sensitivity of the Fully Adjusted Relative Hazard[a] for Heart Failure Subtypes by U.S. EPA Radon Concentration (1993–2024). Shown is a forest plot of hazard ratios (95% confidence intervals) for heart failure with preserved, reduced, or unknown ejection fraction from sensitivity analyses comparing >4 and 2–4 with <2 pCi/L. Estimates are of the association between U.S. EPA radon concentration and heart failure subtype over an attained age time scale obtained from joint Cox proportional hazard models fully adjusted for the covariates listed in Table 2. Points represent the hazard ratios for each exposure category and horizontal lines indicate 95% confidence intervals. The vertical dashed line marks the null value (hazard ratio = 1). EF denotes ejection fraction; P(LRT) likelihood ratio test P value. [a] Tabulated values are hazard ratios (95% confidence intervals). [b] The reference group is <2 pCi/L. [c] Fully adjusted model of the EPA radon concentration-heart failure subtype association from Figure 2. [d] COPD = chronic obstructive pulmonary disease. [e] eGFR = estimated glomerular filtration rate; this analysis was restricted to participants with baseline creatinine measurements. In the referent, COPD-adjusted, and eGFR-adjusted models, global P values for the EPA radon concentration × heart failure classification interactions = .07, .07, and .08.

© 2026 Chen S et al. JAMA Network Open.

eReferences

1. The Women's Health Initiative Study Group. Design of the Women's Health Initiative clinical trial and observational study. *Control Clin Trials.* 1998;19(1):61-109.

2. Anderson GL, Manson J, Wallace R, et al. Implementation of the Women's Health Initiative study design. *Ann Epidemiol*. 2003;13(Suppl 9):S5–S17.

3. Women's Health Initiative. *Annual progress report 2020.* Seattle, WA: Fred Hutchinson Cancer Research Center; 2020. https://www.whi.org/doc/2020-Annual.pdf. Accessed January 26, 2026.

4. U.S. Geological Survey. *Geologic radon potential map of the United States.* U.S. Department of the Interior; 2025. https://www.usgs.gov/data/geologic-radon-potential-map-united-states. Accessed January 26, 2026.

5. Buchheit SF, Collins JM, Anthony KM, et al.; for the Women's Health Initiative Investigators. Radon exposure and incident stroke risk in the Women's Health Initiative. *Neurology.* 2024;102(4):e209143.

6. U.S. Environmental Protection Agency. *EPA map of radon zones: national summary.* Washington, DC: U.S. Environmental Protection Agency; 1993. https://www.epa.gov/radon/epa-map-radon-zones. Accessed January 26, 2026.

7. Lin C, Gelman A, Price PN, Krantz D. Analysis of local decisions using hierarchical modeling, applied to home radon measurement and remediation. *Stat Sci*. 1999;14(3):305-337.

8. Price PN, Nero AV, Revzan KL, et al. *Predicted parameters of radon distributions within counties in the 48 conterminous states.* Berkeley, CA: Lawrence Berkeley National Laboratory; 1996.

9. Li L, Coull BA, Zilli Vieira CL, Koutrakis P. High-resolution national radon maps based on massive indoor measurements in the United States. *Proc Natl Acad Sci U S A*. 2025;122(3):e2408084121.

10. Diez Roux AV, Kiefe CI, Jacobs DR Jr, et al. Area characteristics and individual-level socioeconomic position indicators in three population-based epidemiologic studies. *Ann Epidemiol*. 2001;11(6):395–405.

11. Nayor M, Vasan RS. Preventing heart failure: the role of physical activity. *Curr Opin Cardiol*. 2015;30(5):543–50.

12. Cox DR. Regression models and life-tables. *J R Stat Soc Series B*. 1972;34(2):187–220.

13. Vyas MV, Fang J, Kapral MK, Austin PC. Choice of time-scale in time-to-event analysis: evaluating age-dependent associations. Ann Epidemiol. 2021;62:69-76.

14. Therneau TM. *A package for survival analysis in R.* Version 3.8-3; 2024. https://CRAN.R-project.org/package=survival. Accessed January 26, 2026.

15. Grambsch PM, Therneau TM. Proportional hazards tests and diagnostics based on weighted residuals. *Biometrika.* 1994;81(3):515–26.

16. Choi Y, Kim J, Lee DB, et al. Survival analysis: Part II – applied clinical data. *Korean J Anesthesiol.* 2019;72(5):441–57.

17. Roger VL. Epidemiology of heart failure: a contemporary perspective. *Circ Res.* 2021;128(10):1421–34.

18. Tromp J, Paniagua SMA, Lau ES, et al. Age-dependent associations of risk factors with heart failure: pooled population-based cohort study. *BMJ*. 2021;372:n461.

19. Rothman KJ, Greenland S. *Modern epidemiology.* 2nd ed. Philadelphia, PA: Lippincott-Raven; 1998.

22. Therneau TM, Grambsch PM, Pankratz VS. Penalized survival models and frailty. *J Comput Graph Stat.* 2003;12(1):156–75.

23. Inker LA, Eneanya ND, Coresh J, et al. New creatinine- and cystatin C–based equations to estimate GFR without race. *N Engl J Med.* 2021;385(19):1737–49.

24. Van Buuren S, Groothuis-Oudshoorn K. mice: multivariate imputation by chained equations in R. J Stat Softw. 2011;45(3):1-67. https://www.jstatsoft.org/v45/i03/. Accessed January 26, 2026.

25. Rubin DB. *Multiple imputation for nonresponse in surveys.* New York, NY: John Wiley & Sons; 1987.

© 2026 Chen S et al. JAMA Network Open.

26. Xue X, Kim MY, Gaudet MM, et al. A comparison of the polytomous logistic regression and joint Cox proportional hazards models for evaluating multiple disease subtypes in prospective cohort studies. *Cancer Epidemiol Biomarkers Prev*. 2013;22(2):275–85.

27. Fine JP, Gray RJ. A proportional hazards model for the subdistribution of a competing risk. *J Am Stat Assoc.* 1999;94(446):496–509.

28. Scheike TH, Zhang MJ. Analyzing competing risk data using the R timereg package. *J Stat Softw.* 2011;38(2):1–15.

29. Harrell FE Jr. *Regression modeling strategies: with applications to linear models, logistic and ordinal regression, and survival analysis.* 2nd ed. New York, NY: Springer; 2015.

© 2026 Chen S et al. JAMA Network Open.
