# Supplementary material for: Indoor Radon and Incident Heart Failure Among Postmenopausal Women
Source: JAMA Netw Open. 2026 Nov 5;9(11):e2642391. doi: 10.1001/jamanetworkopen.2026.42391 (PMC13741163; doi:10.1001/jamanetworkopen.2026.42391)
Supplement: Supplementary file 2 [file jamanetwopen-e2642391-s002.pdf]

Data Sharing Statement

Chen. Indoor Radon and Incident Heart Failure Among Postmenopausal Women. *JAMA Netw Open*. Published November 05, 2026. doi:10.1001/jamanetworkopen.2026.42391

Data
Data available: Yes
Data types: Deidentified participant data, Data dictionary
How to access data: https://www.whi.org/get-started https://www.whi.org/propose-a-paper https://www.whi.org/plan-a-study
When available: With publication

Supporting Documents
Document types: None

Additional Information
Who can access the data: Per WHI policy (https://www.whi.org/doc/PP-policy.pdf; https://www.whi.org/doc/AS+Policy.pdf), investigators with an approved manuscript or ancillary study proposal.
Types of analyses: All those allowable under the above policies.
Mechanisms of data availability: After approval of a proposal and execution of data use agreement.
Any additional restrictions: None
